# Exploring the Surface Potential of Recycled Polyethylene Terephthalate Composite Supports on the Collagen Contamination Level

**DOI:** 10.3390/polym15030776

**Published:** 2023-02-03

**Authors:** Elena-Luiza Epure, Florina Daniela Cojocaru, Mihaela Aradoaei, Romeo Cristian Ciobanu, Gianina Dodi

**Affiliations:** 1 Faculty of Chemical Engineering and Environmental Protection, Gheorghe Asachi Technical University, Mangeron Bd., 700454 Iasi, Romania; 2 Advanced Research and Development Center for Experimental Medicine, Grigore T. Popa University of Medicine and Pharmacy of Iasi, 9-13 M. Kogalniceanu Street, 700454 Iasi, Romania; 3 Faculty of Medical Bioengineering, Grigore T. Popa University of Medicine and Pharmacy of Iasi, 9-13 M. Kogalniceanu Street, 700454 Iasi, Romania; 4 Electrical Engineering Faculty, Gheorghe Asachi Technical University of Iasi, 67 Prof. Dimitrie Mangeron Bd., 700050 Iasi, Romania; 5 ALL GREEN SRL, 8 G. Cosbuc Street, 700470 Iasi, Romania

**Keywords:** PET, HDPE, PP, recycling, contact angle, spectroscopy

## Abstract

With a significant number of features (namely being multipurpose, inexpensive and durable), thermoplastic polymers, most often named plastics, are part of our daily routine, with an increasing production over the last decade. Among them, polyethylene terephthalate (PET), high-density polyethylene (HDPE) and polypropylene (PP) are distinguished as the five most commonly used plastics in various fields, mainly in the packaging industry. Even if it is difficult to imagine the world without plastics, the boosted plastic assembly comes with huge plastic waste, creating a number of challenges, as the most important threat for our environment, but also opportunities for recycling. Currently, a special attention is dedicated on how to improve the current recycling methods or to find new ones, since the quality of recycled plastics and potential chemical or biological contaminations are two problematic aspects. Understanding the properties of each thermoplastic polymer and the interaction with possible contaminants may be the key for an efficient recycling process. The aim of this paper was to evaluate the surface behaviour of different composite supports based on recycled PET before and after interaction with collagen (used as a biological contaminant). The surface contamination bias of PET supports was studied through different techniques: scanning electron microscopy (SEM), water uptake through swelling studies, contact angle measurements and attenuated total reflection–Fourier transform infrared spectroscopy (ATR-FTIR).

## 1. Introduction

It is well known that plastic alternatives are a must, and different support initiatives to develop them are under great expansion; however, the reality is that plastic materials and products are now indispensable to modern society [1], with a global production estimated at over 390 million tons in 2021 and an annual increase of four percent [2].

Polyethylene terephthalate (PET), high and low-density polyethylene (HDPE, LDHE), polypropylene (PP), polyvinyl chloride (PVC) and polystyrene (PS) are considered the most encountered and cost-effective plastics used in the production and also in recycling [3]. 

PET is the most common semi-crystalline thermoplastic polymer of the polyester family, known for valuable features such as easy processing capacity, low cost, lack of toxicity, high stiffness, excellent mechanical strength and chemical resistance [4]. Part of the polyethylene family, HDPE occupies second place in the most commonly used plastics list due to its good chemical resistance ability, along with high flexibility, weatherproof properties, good low temperature toughness and low cost [5]. PP, a partially crystalline and non-polar polymer that belongs to the polyolefins group, is located at the fifth place as one of the most durable types of plastic [6,7].

While the uses and production of synthetic polymers have increased exponentially over the past 50 years, the main issue is that they are derived from non-renewable resources and the costs associated with their disposal are dramatically increasing [8]. Today, only 9% of plastic waste is recycled, whereas 22% is mismanaged [9]. Considering the global value of recycled plastic, different approaches to waste management are necessary to improve the circularity of plastics. 

Although the processability of different kinds of PET, PP and HDPE packaging types varies considerably, current recycling of mixed composite waste will not technically facilitate recycling into new products. This underlines the importance of composite polymer waste homogeneity when sent to recycling. Such uniformity may be achieved through product design management, accounting for polymer properties and recyclability.

Although PET is the leader in recycling (100% recyclable [10]), intense research is still ongoing to find new resolutions for using safe uncontaminated recycled plastic [11]. In the report of Awaja and Pavel [12], the level of contamination was considered the most important factor in the reuse of recycled PET plastic. The authors did not confirm the dependence of different contaminates, such as acid-producing contaminants, water, colouring contaminants, bacteria, acetaldehyde, detergents, fuel, pesticides, etc. [11] on the mechanical properties but it is clear that contamination is the major cause of deterioration of its physical and chemical properties during re-processing [13].

In a more recent study, Eriksen et al. [14] also pointed out that the plastics from household waste are a contaminated resource that leads to recycled plastic with reduced quality and limited potential for closed-loop recycling. They demonstrated that PET, PE and PP recycling represent different challenges, one of them being the contamination process, highlighting the importance of effective decontamination approaches in order to efficiently restore the polymer chains based on reliable methods that determine and quantify the contaminant. 

Collagen is the main structural protein produced by the body and has an essential role in the mechanical features, organisation and shape of tissues [15]. Due to its biological functions, collagen has numerous applications in human medicine, namely in tissue bioengineering, implant matrices, burn dressings, vascular prostheses, membranes, haemostatic foams, transport systems for steroids, antibiotics and other drugs, etc. Also, collagen is a by-product of meat and leather production and is used in food industry and for clothing and footwear and other goods manufacturing. All these products could be found on the packing materials, which can further enter into the recycling process; therefore, in this study, collagen was used as an artificial contaminant mimicking proteins’ structures.

In the context of finding efficient solutions to identify different contaminants of the recycled plastics, the main aim of the present work is to evaluate the behaviour of different recycled PET composite surfaces before and after artificial contamination with collagen solution. The performance was assessed using well-known approaches, from morphology using a scanning electron microscope, water uptake through swelling studies and contact angle measurements and functional group identification by attenuated total reflection FTIR spectroscopy.

## 2. Materials and Methods

### 2.1. Materials

The composite supports were obtained by ALL GREEN SRL Iasi, Romania following a protocol partially described in our previous published paper [16]. Briefly, recycled PET (obtained in-house from waste plastic bottles), PP (pellets, melt mass-flow rate—MFR at 230 °C/2.16 kg: 8.0 g/10 min, Tipplen H 318), HDPE (pellets, MFR at 190 °C/2.16 kg: 6.5 g/10 min, 71% crystallinity grade, Tipelin 1100J), were purchased from MOL Petrochemicals Co. Ltd., Tiszaújváros, Hungary, aluminium—Al nano-powder, 800 nm (purity: 99.995%, specific surface area: 15–20 m^2^/g), iron—Fe cubic nano-powder, 790 nm (purity: 99.55%, density: 7.9 g/cm^3^) from Nanografi LTD. STI, Ankara, Turkey and collagen A solution (1 mg/mL in water, Biochrom, Berlin, Germany).

### 2.2. Recycled PET Supports Preparation and Evaluation

#### 2.2.1. Synthesis Procedure

The recycled PET composite materials were obtained using an injection-moulding machine (Dr. Boy, Neustadt, Wied, Germany). The hybrid substrate contains several components, namely, PET as the main constituent of all the materials in different percentages (100, 95, 92, 70, 66.5 or 64.5%), combined or not with PP/HDPE (30, 28.5 and 27.7%) and Al/Fe nano-powders (8, 5%). All the elements are reported as the percentage by weight from the whole quantity of dried material. The composition for each substrate used in the manufacturing process is displayed in Table 1. 

The temperature regime and processing conditions used on the injection-moulding machine for each of the codified experimental model is presented in Table 2.

The obtained hydrostatic density (HD), measured using a Mettler Toledo™ analytical balance with density kit (Columbus, OH, USA), was indicated in Table 1 for each hybrid substrate as the HD ± standard deviation of three independent measurements.

#### 2.2.2. Water Uptake Capacity of PET-Based Substrates

Water uptake of injection moulded PET-based samples was determined according to ISO 62:2008 [17] using approximately 85 mg specimens from each material, completely immersed in distilled water and maintained at 22 °C and 41% humidity for a long period, from 7 and up to 50 days. The water/swelling degree percentage was calculated using the following equation:

$$Q\;\left(\%\right)=\frac{x_{2}-x_{1}}{x_{1}}\times 100$$

where, *x*_1_ is the initial weight of the sample and *x*_2_ is the weight of the swelled sample.

The swelling test was conducted according to ISO 62:2008 and is extended until a quantity of 30% of the mass of the sample is absorbed. Such a high percentage is not the case with plastic materials, therefore the exposure was limited to a reasonable maximum duration of 50 days, and the water uptake capacity was calculated for that duration.

#### 2.2.3. PET-Based Substrate Surface Morphology 

The morphologies of the PET support surfaces were analysed using a scanning electron microscope (Zeiss, White Plains, NY, USA) with an Everhart-Thornley-type secondary electron detector and Faraday cage. The dried sheets of PET composites were gold sputtered for producing electric conductivity. Photomicrographs of PET pellets were viewed in the vacuum condition at 1 or 2 kV. 

### 2.3. Collagen Artificial Contamination of PET-Composite Pellets 

A total of 50 μL collagen solution of 1 mg/mL was added in droplets on clean, decontaminated pellet samples of about 5–10 mm and 80–110 μm thickness and incubated at room temperature for 50 days. The collagen solution was added daily for 50 days in order to mimic the long-term scenarios but also to avoid the formation of microorganisms in the solution. 

#### 2.3.1. ATR-IR Spectra before and after Collagen Deposition

Functional groups of the specimens’ samples were analysed using a Nicolet Summit Pro FTIR spectrometer with diamond Everest ATR accessory (Thermo Scientific, Waltham, MA, USA). A wavelength of 4000–400 cm^−1^ range was used and the chemical groups available onto the surface plates were identified by their peaks using IR Omnic software (16 scans, 4 cm^−1^). Each pellet was analysed before and after collagen deposition at different time-points, namely, 2 h every day in the first 7 days and then at 17, 21, 25 and 50 days, denoted in the manuscript as T_7-50_.

#### 2.3.2. Contact Angle Measurements

Static water contact angles measurements were performed on a drop shape analyser (Kruss Easy Drop goniometer, Hamburg, Germany) in an ambient environment of 25 °C temperature and 16% relative humidity. The water droplet volume was set to ~2 μL. After each measurement, the pellets were dried in air and placed again for analysis. Each measurement was repeated 4 times for a better statistical significance. The error bars resulted from the standard deviation based on the measurement series. The contact angle at the three-phase contact point was calculated using Drop Shape Analysis (DSA) version 1.90.0.14 software during the 30 s onto the simple pellets and also on the collagen deposited ones. The right and left contact angles were averaged.

## 3. Results and Discussions

### 3.1. Composite Materials Based on Recycled PET and Their Properties

Three classes of PET-based composites were prepared via the injection moulding procedure starting from the raw PET support (codified M1). These constructed pellets were categorized based on their composition, namely:

- composite materials containing Al or Fe nano-powders;
- composite materials containing PP or HDPE components;
- composite materials containing PP or HDPE components and Al or Fe nano-powders.

The nano-powders supplements that introduce metal into the plastic composition are related to the initiative to obtain new packaging formulas for food and drugs, with greater resistance to light/UV radiation and limiting the activity of chemical and bacterial contamination. If the packaging comes from recycled sources, this kind of packaging can be additionally treated for decontamination by exposure to microwave radiation, where the metal powders become nano-energy concentrators.

The addition of metal nano-powders as reinforcements into the obtained composite materials influenced the processing behaviour of the pellets, namely the ones containing Al nano-powders, M2, M3, M7, M8, M12 and M13 are easier to process than those containing Fe nano-powders (M4, M5, M9, M10, M14 and M15). This was due to the fact that Al is a soft metal and allows easy embedding into the polymer matrix, which also induces better homogenization. The data from the literature presents various kinds of fillers, additives and reinforcements that are introduced into the polymer matrix to improve either thermal and electrical conductivity or mechanical properties, as briefly detailed below. In a recent study, Anis et al. [18] described the first case where the addition of Al particles to PET simultaneously increased the modulus and impact resistance. The results displayed that Al powders can not only act as toughening agents without decreasing the modulus and strength but also as nucleating agents; however, they are insufficient to make PET crystallize. Thus, the Al–PET composites have an untypical pattern where the modulus increased, the strength did not drop and the impact resistance increased at all compositions. When Fe nano-powder is used as reinforcement for HDPE, PP or PS composite materials [19], the mechanical properties are customized, namely reduced Izod impact strength, yield and tensile strength, % elongation and increased modulus of elasticity. These indicators were not determined for the obtained PET supports and as such do not make the subject of this paper. 

PP and HDPE incorporation into the obtained PET-based pellets also influenced the material properties and design; the ones containing PP were more flexible than those containing HDPE. 

The slight variations in the hydrostatic density, as presented in Table 1, were guided by all components. Even if the used polymers have different density values: 0.96–1.45 g/cm^3^ for PET, 0.02–0.83 g/cm^3^ for PP and 0.94–0.98 g/cm^3^ for HDPE [20] and density is a key factor that differentiates HDPE from PP, the obtained values are comparable for the samples based only on polymers. A higher HD was obtained for the sample containing PET–PP–Fe nano-powder (M10), which indicates an increase in the crystalline phase, and the lowest HD was from the composite comprised only of PET–PP (M6), probably due to the higher density of Fe nano-powder as compared with Al nano-powder (almost one third). 

SEM analysis was undertaken to determine how the morphology of the PET matrix changed with the PP, HDPE, Al and Fe nano-powder contents. SEM was used to obtain the morphology of pellet injection-moulded samples before collagen artificial contamination, thus providing valuable information. As can be seen in Figure 1, Figure 2, Figure 3, Figure 4, Figure 5, Figure 6, Figure 7, Figure 8, Figure 9, Figure 10, Figure 11, Figure 12, Figure 13, Figure 14 and Figure 15, even if all samples present a good microscopic structure, important differences should be highlighted.

**Figure 1:**
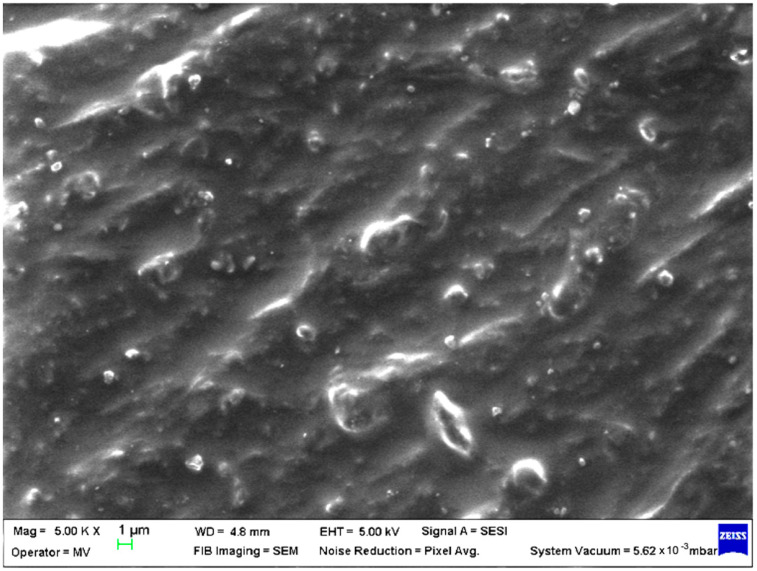
SEM micrographs of PET (M1) sample at T_0_ (1 µm scale).

The recycled PET sample, as presented in Figure 1 (M1), is not featureless at the 1 µm scale. As for pure PET samples [18], it presents irregularities mainly since the dispersion and distribution of the PET phase is not very uniform.

**Figure 2:**
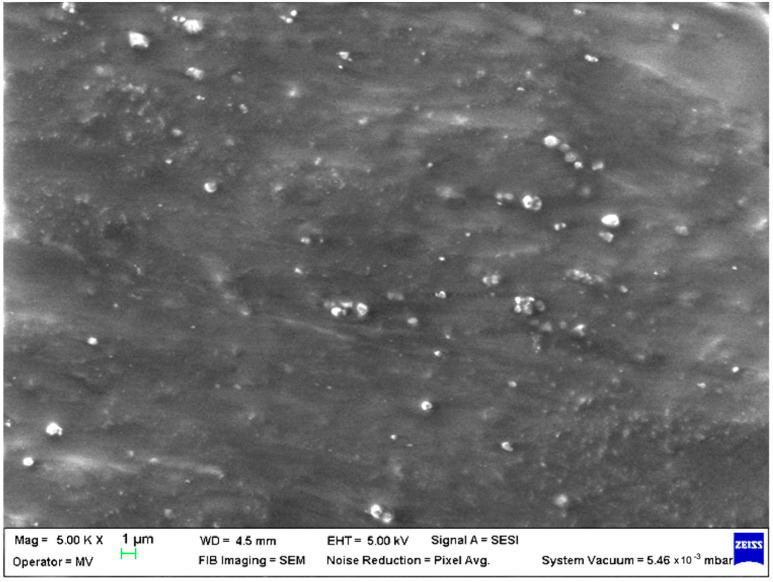
SEM micrographs of PET–Al composite (M2) sample at T_0_ (1 µm scale).

The addition of 5% Al nano-powder into the PET phase (sample M2, Figure 2) produces a relatively smoother surface but with some small aggregates that are either embedded under a coating of PET or protruded.

**Figure 3:**
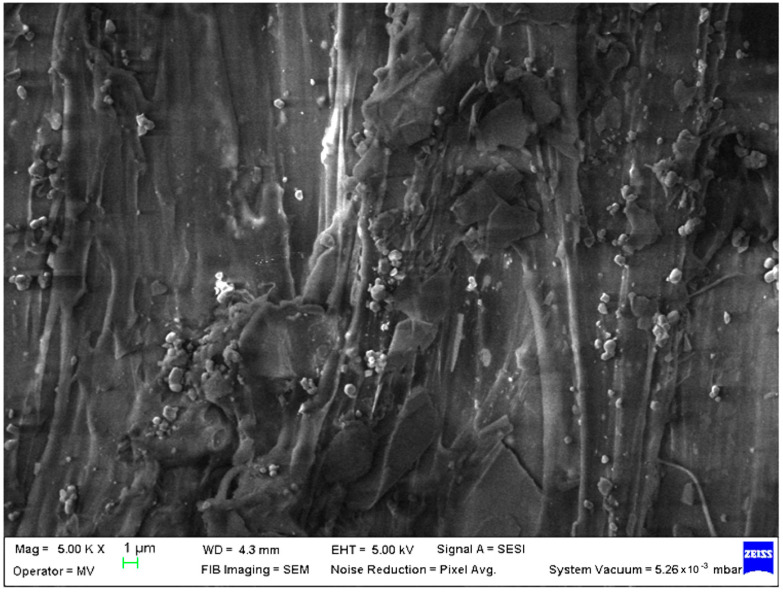
SEM micrographs of PET–Al composites (M3) sample at T_0_ (1 µm scale).

As the Al nano-powder loading increased beyond 5% to 8%, Figure 3 (sample M3) shows larger aggregates projected from the PET coating with a slightly deformed texture. 

**Figure 4:**
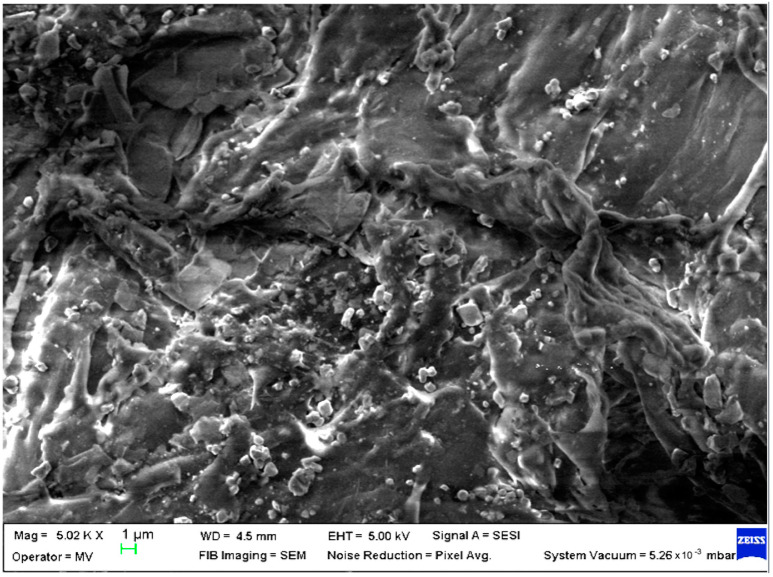
SEM micrographs of PET–Fe composite (M4) sample at T_0_ (1 µm scale).

**Figure 5:**
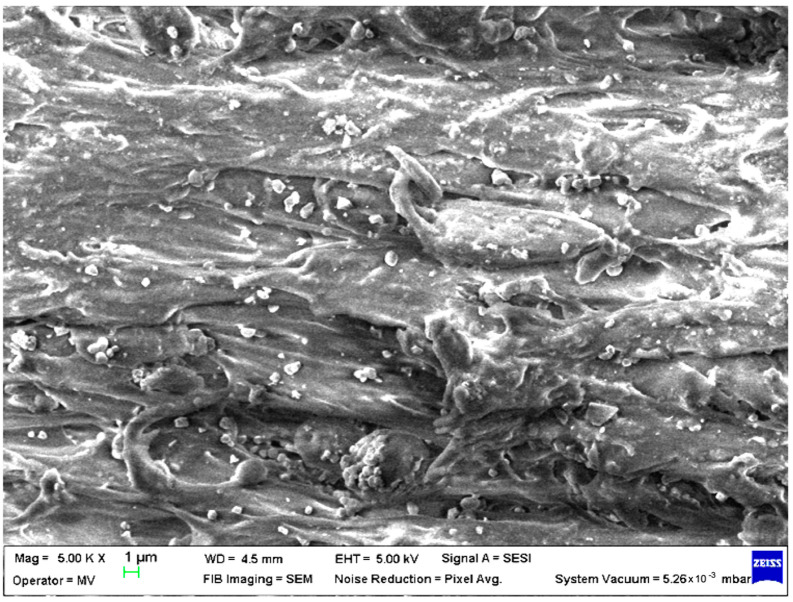
SEM micrographs of PET–Fe composite (M5) sample at T_0_ (1 µm scale).

In sample M4, containing PET and 5% Fe- nano-powder, the deformation pattern tended to change from smooth to brittle, with the appearance of some voids in the fracture surface due to the dropout of Fe particles (Figure 4). The same morphology is also observed for sample M5 (Figure 5) where the Fe nano-powder content increased up to 8%.

**Figure 6:**
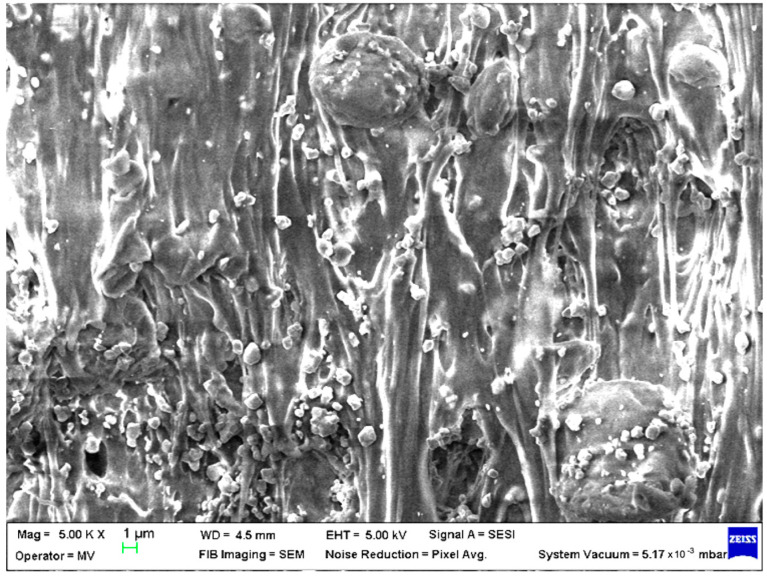
SEM micrographs of PET–PP composites (M6) sample at T_0_ (1 µm scale).

The SEM micrograph of a PET–PP composite pellet (sample M6) revealed a highly open structure with multiple irregularities due to the PP fibres (Figure 6). 

**Figure 7:**
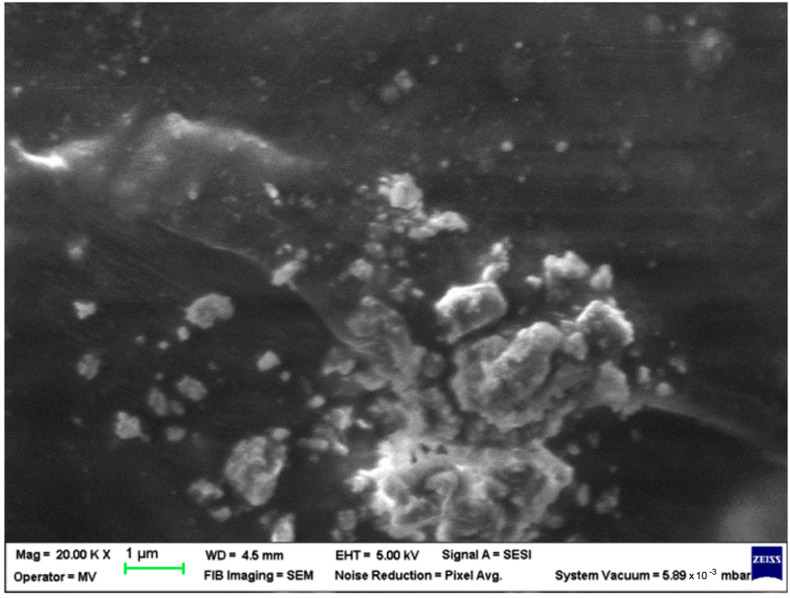
SEM micrographs of PET–PP–Al composite (M7) sample at T_0_ (1 µm scale).

**Figure 8:**
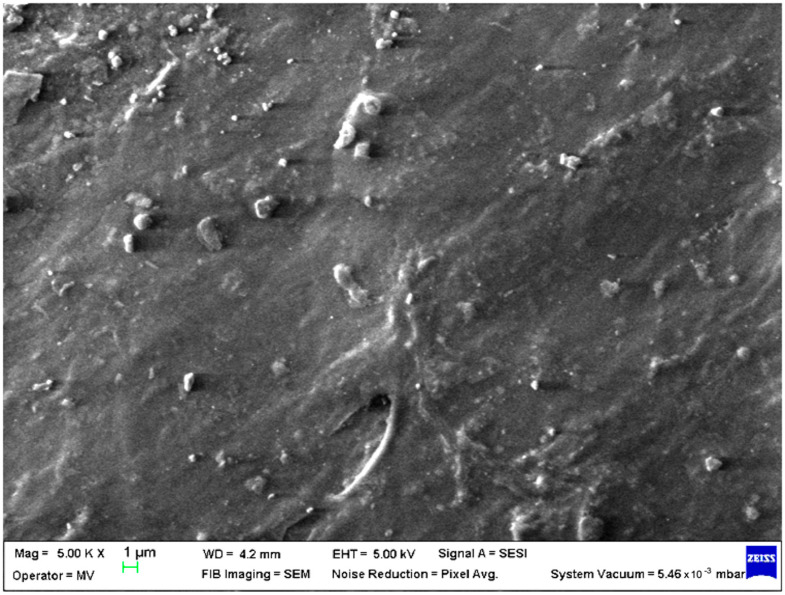
SEM micrographs of PET–PP–Al composite (M8) sample at T_0_ (1 µm scale).

The surface morphology of the PET–PP composite at different loading percentages of the Al nano-powder is shown in Figure 7 (sample M7) and in Figure 8 (sample M8) displayed a more compact structure compared with the bare pellet, with large and agglomerated aggregates for low percentage and small lumps and few drop-outs for 8% metal particles.

**Figure 9:**
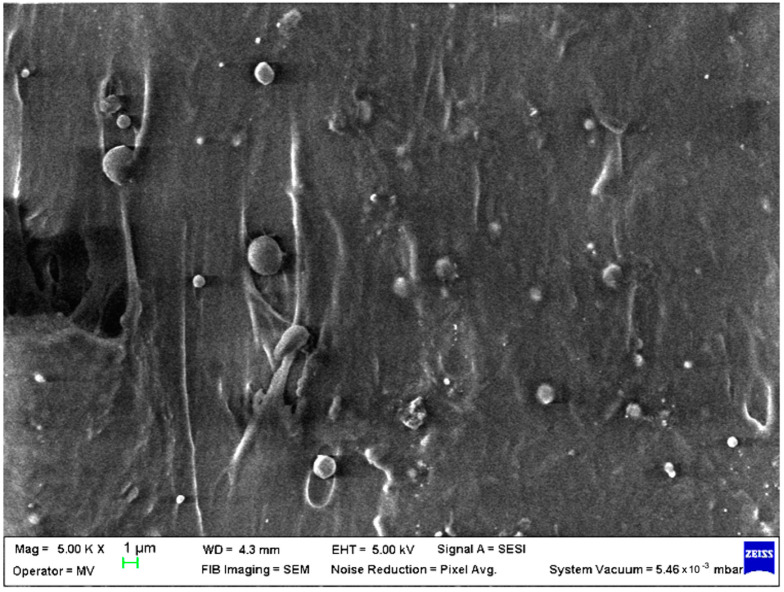
SEM micrographs of PET–PP–Fe composite (M9) sample at T_0_ (1 µm scale).

**Figure 10:**
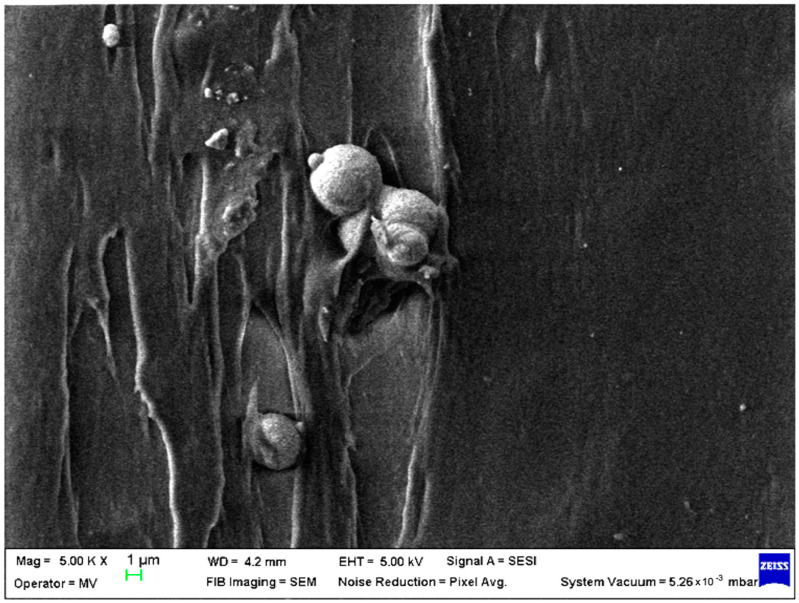
SEM micrographs of PET–PP–Fe composite (M10) sample at T_0_ (1 µm scale).

The Fe nano-powder content had the same effect on the surface morphology for PET–PP composite materials, the micrographs showing a neat structure with well-embedded Fe particles and protuberances (samples M9 and M10; Figure 9 and Figure 10). 

**Figure 11:**
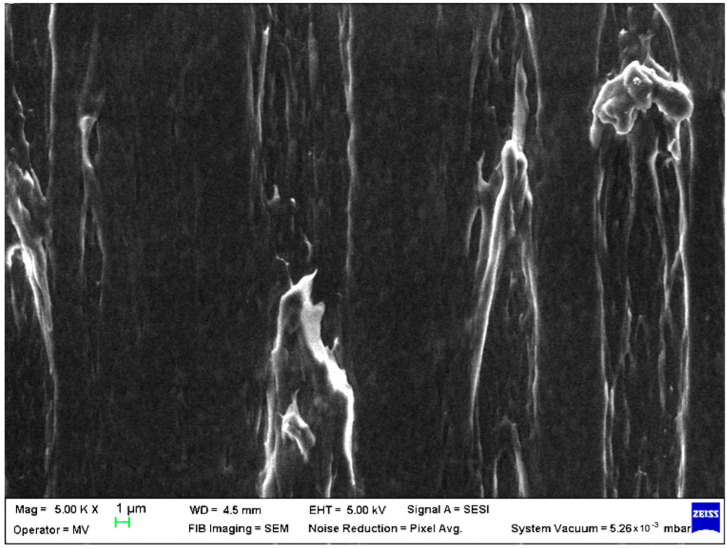
SEM micrographs of PET–HDPE composite (M11) sample at T_0_ (1 µm scale).

The surface morphology changes with the addition of HDPE in the PET matrix, leaving a smooth pattern with aligned areas where the fibres overlapped forming oriented lumps; this indicates a good interfacial interaction between the HDPE and the PET matrix (Figure 11).

**Figure 12:**
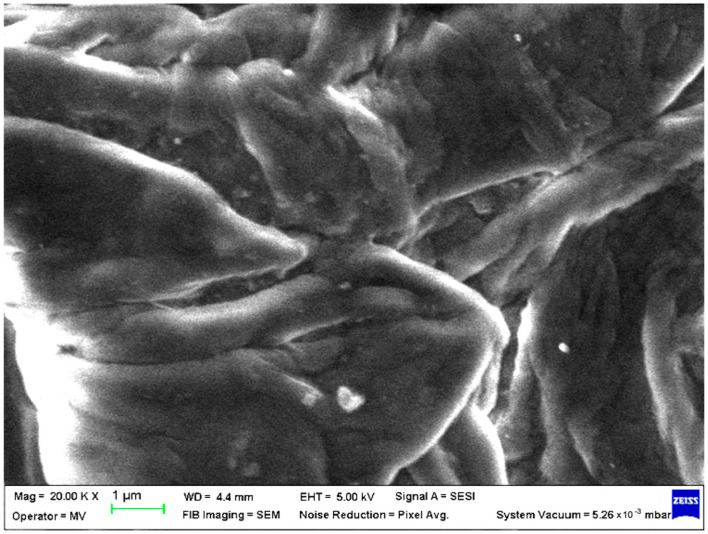
SEM micrographs of PET–HDPE–Al composite (M12) sample at T_0_ (1 µm scale).

The PET–HDPE composite with Al particles (sample M12, Figure 12) displays an arrangement with interconnected fibres that cannot contribute to water permeation.

The causes of these imperfections could be multiple air cavities or residual impurities in the recycled PET, PP/HDPE or Al/Fe particles with a tendency to agglomerate.

**Figure 13:**
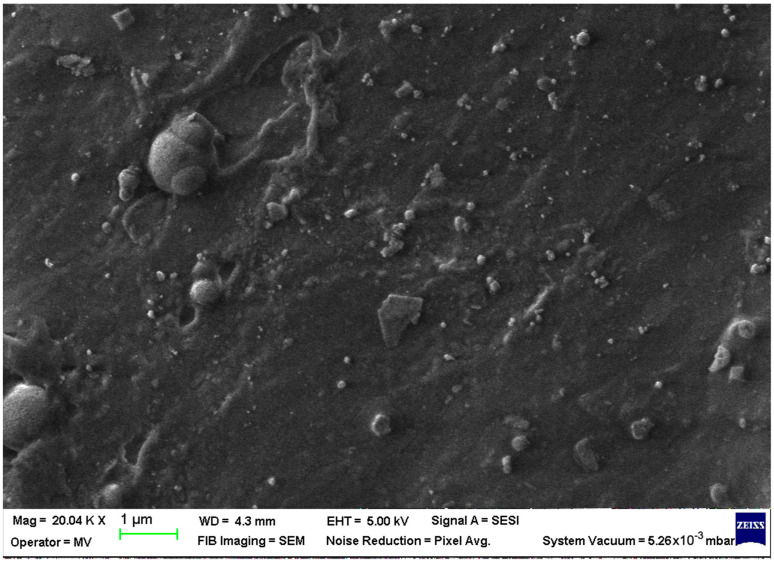
SEM micrographs of PET–HDPE–Al composite (M13) sample at T_0_ (1 µm scale).

The Al nano-powder content of 8% had a different effect on the surface morphology for PET–HDPE composite material, when compared with an Al content of 5%, the micrographs display an ordered organisation with well-embedded Al particles and protuberances (sample M13; Figure 13). 

**Figure 14:**
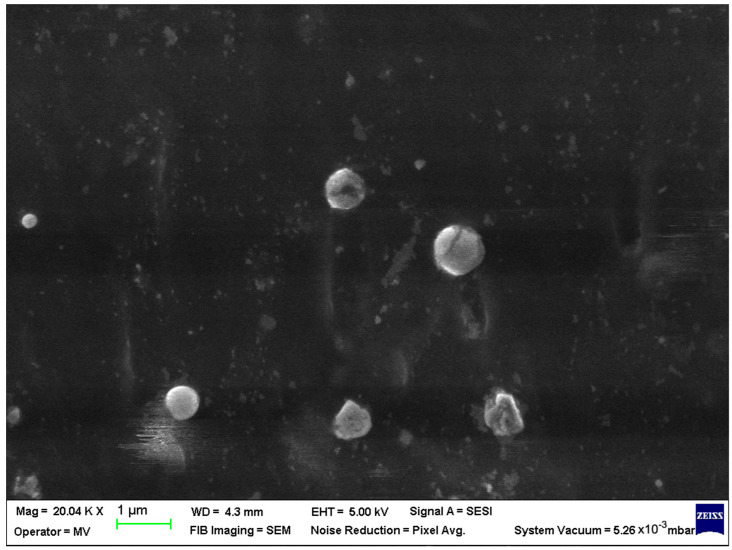
SEM micrographs of PET–HDPE–Fe composite (M14) sample at T_0_ (1 µm scale).

**Figure 15:**
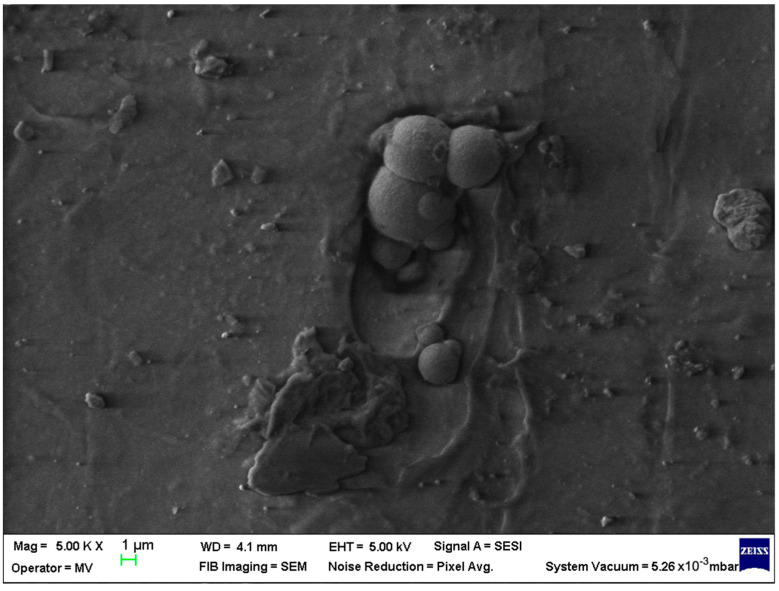
SEM micrograph of PET–HDPE–Fe composite (M15) sample at T_0_ (1 µm scale).

The HDPE and PET smooth pattern surface of samples M14 (Figure 14) and M15 (Figure 15) is disordered by the Fe nano-powder content, showing different lumps, more pronounced at a higher concentration. 

### 3.2. Surface Potential of Recycled PET Composite Supports on the Collagen Contamination Level

**Figure 16:**
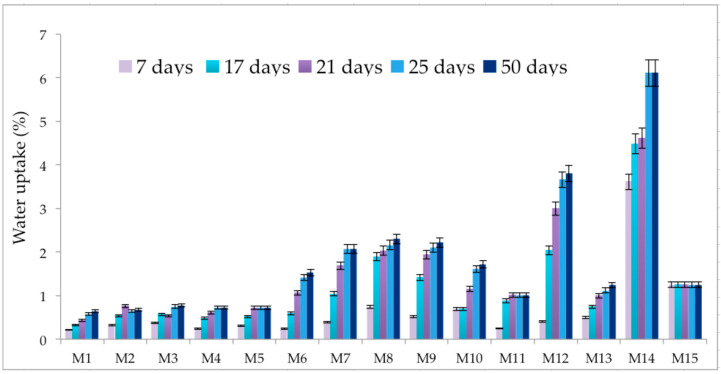
Wettability of the surface at different time-points.

Water immersion behaviour (Figure 16 and Table 3) was tested for all samples for 50 days in order to determine the wettability of the surface by measuring the amount of liquid that the material can absorb when immersed in that medium. As observed in Figure 6 and Table 3, the PET pellet surface remains nearly intact in contact with water since PET is a semi-crystalline polymer that incorporates an ester group, and the values obtained are indicative of the low polarity of the polymer [21]. The non-swelling potential of the PET sample is correlated with its hydrophobic character as observed in Figure 7 and Table 4 and has a value similar to that of native PET of about 91.3° [22].

**Figure 17:**
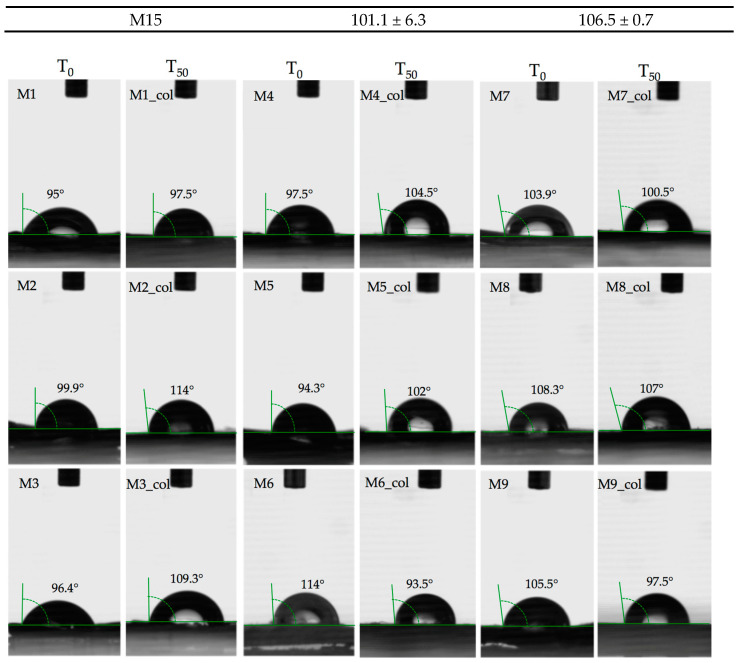
Contact angle of PET composites before and after collagen accumulation at 50 days (T_50_).

The slight hydrophobic behaviour permitted the deposition of collagen chains into the PET irregularities found on the surface, thus moving the contact angle by 2 degrees, as observed in Table 4 and Figure 17. 

The addition of PP and HDPE components into the composite structure determined the same behaviour upon complete immersion in water since both polymers are semicrystalline with non-wettable surfaces. As expected, the low values of the swelling degree obtained after 7 days of complete immersion in water, increased in time, as an outcome of the changes in surface wettability of the polymeric materials immersed in an aqueous medium for an extended period [23].

As observed in Figure 17, the hydrophilic PET–PP composite material had a higher degree of hydrophobicity of about 114° since the native contact angle of PP is according to literature 104.9°, higher than that of bare PET. The surface morphology, namely pillar-like features with uniform periodicity and depth as observed by SEM, could induce an increase in the contact angle. These micro-features induced an increase in the collagen penetration, demonstrated by the contact angle after 50 days of complete immersion being about 93.5°. 

Even if the swelling degree of the samples containing Al nano-powder (sample M7 and M8) is higher than bare PET (around 2%), the hydrophobic character given by the PP blends is maintained and the collagen spreads in a small proportion onto the surface structure, thus the contact angle is around 100° (Figure 17, Table 4). 

**Figure 18:**
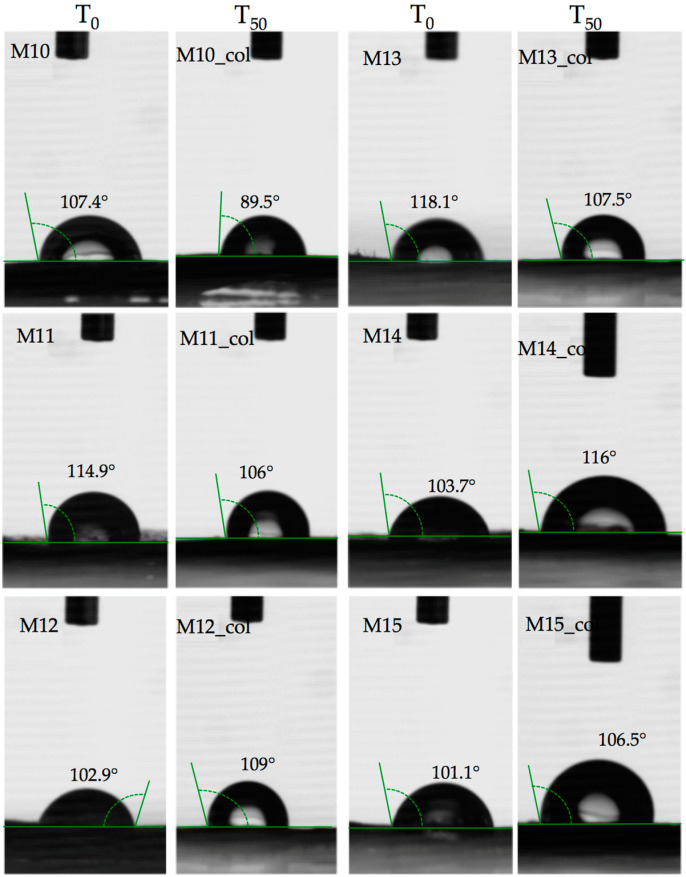
Contact angle of PET composites before and after collagen accumulation at 50 days (T_50_).

A different behaviour was observed for the polymer matrices based on PET–PP and Fe nano-powder content (samples M9 and M10; Figure 17 and Figure 18). Before the contamination with collagen solution, the non-wettable surface was defined by high contact angles of about 105° and 107°; however, due to the surface pattern of the blends during the manufacturing process with obvious protuberances, the collagen managed to form bridges and the contact angle decreased up to 90°. 

The influence of PP into the matrix composition is demonstrated by the contact angles of the PET–Al nano-powder pellets being about 90°, relatively similar to the native PET. 

The wettability of the PET–HDPE composite (sample M11: Figure 18 and Table 4) is higher, about 114.9°, even if the two polymers have similar contact angles around 95°, mainly since the surface is smooth and the blends are uniformly distributed. In this context, the water molecules could not penetrate inside the blends, hence the collagen is deposited probably in a small amount, thus reducing the contact angle to 106°. 

The Al nano-powder probably did not influence the surface behaviour of the PET–HDPE composite materials (samples M12 and M13, Figure 18) since the contact angle values were maintained in the same range as the bare material. Also, the texture pattern of the pellets permitted collagen deposition but in a small percentage as shown by the values of the contact angle, even if the swelling degrees were relatively increased.

A similar trend was also observed for the PET–HDPE surfaces containing Fe nano-powders (samples M14 and M15) due to the line-like pattern of the surfaces as observed in the micrographs. As expected, the contact angle signified a hydrophobic surface on the addition of HDPE and of the Fe particles. The presence of metals in a polymer matrix can induce plasticising effects by interpenetrating the polymer network, thus reducing the packing of polymers. At the macro level, this fact will translate into an increase in the contact angle [24]. Another not negligible aspect is the incompatibility between the polymers, which can lead to a separation on a microscopic scale.

### 3.3. FTIR-ATR Analysis

The ATR-IR spectrum was analysed to determine the changes in the chemical groups on the pellets’ surfaces and the ability of different recycled plastics to retain the contamination with proteins.

**Figure 19:**
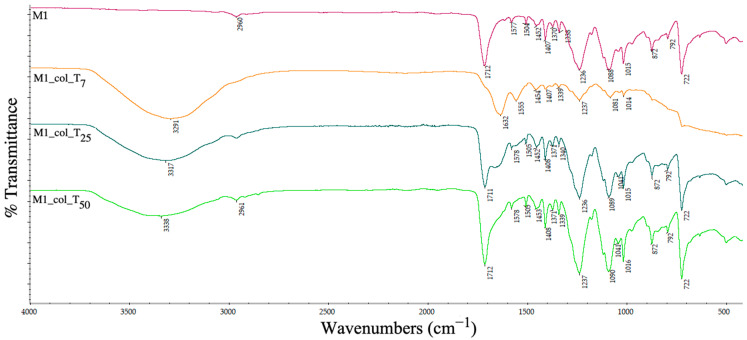
ATR spectra of PET pellets before and after collagen deposition at days 7 (T_7_), 25 (T_25_) and 50 (T_50_).

Figure 19 shows the FTIR-ATR spectrum of the PET (sample M1). A peak of 2960 cm^−1^ indicated the presence of the aliphatic C–H symmetrical bond stretching. At 1712 cm^−1^ and 1407 cm^−1^, an ester carbonyl bond and C–O group deformation of the O–H group were observed. A peak at 1236 cm^−1^ indicated the terephthalate group, at 1088 cm^−1^ the methylene group, at 1015 cm^−1^ the vibrations of the ester C–O bond, at 872 cm^−1^ the presence of the aromatic rings and at 722 cm^−1^ the polar ester groups interaction with benzene rings. According to the data shown in Figure 9 and confirmed by other studies [25], the pellet was composed of PET material.

In the ATR-FTIR spectrum with collagen used as a protein contaminant, the main vibration bands that were identified were: 3289 cm^−1^ and 3260 cm^−1^ attributed to the stretching vibrations of the OH and NH bonds of amide A and 2921 and 2852 cm^−1^ attributed to asymmetric and symmetric stretching of CH_2_ [26]. Absorption of amides I and II were observed at 1630, 1634 and 1536 cm^−1^. The absorption bands at 1447, 1334 and 1234 cm^−1^ can be assigned to the groups δ (CH2), δ (CH3), ν (C–N) and δ (N–H) [27].

The presence of collagen is observed at all time-points on PET sample, with the appearance of new peaks in the 320 cm^−1^ region and at 1632 cm^−1^ and a gradual increase in time of the specific peak intensities, indicating that the contamination level becomes higher as the plastic remains in the immersion environment.

**Figure 20:**
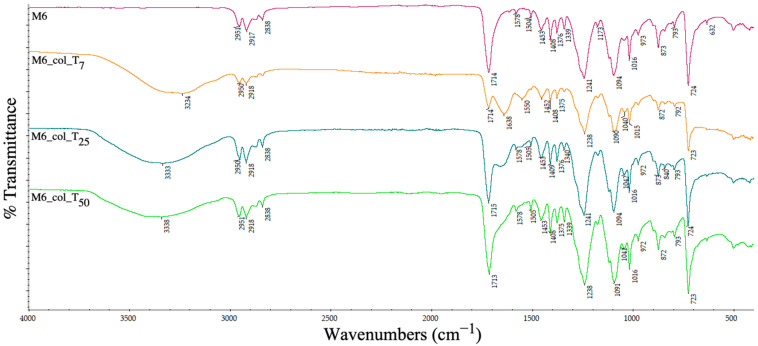
ATR spectra of PET–PP composite pellets before and after collagen deposition at days 7 (T_7_), 25 (T_25_) and 50 (T_50_).

Figure 20 displays, besides the PET specific bands, the additional representative absorption signals for the PP (sample M6) namely: 2917 cm^−1^ for the asymmetric stretching of –CH_2_, 2838 cm^−1^ for the symmetric stretching of the methyl group (-CH_3_) and sharper peaks at 1453 cm^−1^ and 1376 cm^−1^ attributed to –CH_2_- and –CH_3_ bending. All these bands are observed also in samples M7 and M9, since both contain PP.

At 7, 25 and 50 days after the contamination of the PET–PP-based supports (Figure 20), a relatively greater intensification of the absorption bands from the collagen structure is observed due to surface’s ability to retain hydrophilic molecules, as described in the previous section. 

**Figure 21:**
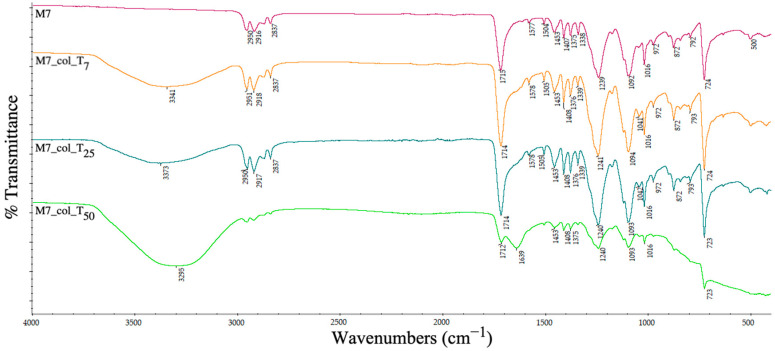
ATR spectra of PET–PP–Al nano-power composite pellets before and after collagen deposition at days 7 (T_7_), 25 (T_25_) and 50 (T_50_).

The existence of Al nano-powder into the recycled PET–PP (5% for sample M7, Figure 21) composition was detected by the weak peaks characteristic of the Al–O stretching and O–Al–O bond in the range of 500–1000 cm^−1^ [28], clear peaks at 500 cm^−1^ and 972 cm^−1^ and other peaks that are overlapping with the ones of the main polymers. Collagen contamination was clearly seen after 50 days of complete immersion, with the appearance of OH bonds (explained by the relatively increased swelling degree) at 3295 cm^−1^ and of amide I at 1639 cm^−1^.

**Figure 22:**
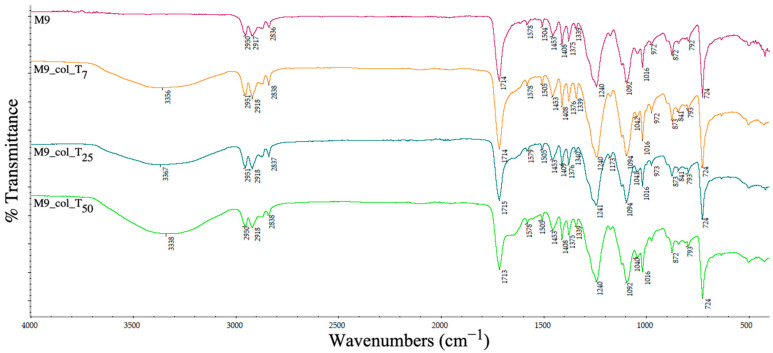
ATR spectra of PET–PP–Fe nano-power embedded pellets before and after collagen deposition at days 7 (T_7_), 25 (T_25_) and 50 (T_50_).

The metal (iron) oxygen stretching vibrational mode was relatively observed in the region of 500–600 cm^−1^, specific for the sample containing 5% Fe nano-powder (sample M9; Figure 22). After collagen bonding with the matrix surface, the bonding in the region 1600 cm^−1^ ascribed to the amide moiety is separating from the ester carbonyl group. The general range of 3300 cm^−1^ may be assigned to the OH bonding stretching vibrational modes for water from the collagen solution in accordance with the swelling degree.

**Figure 23:**
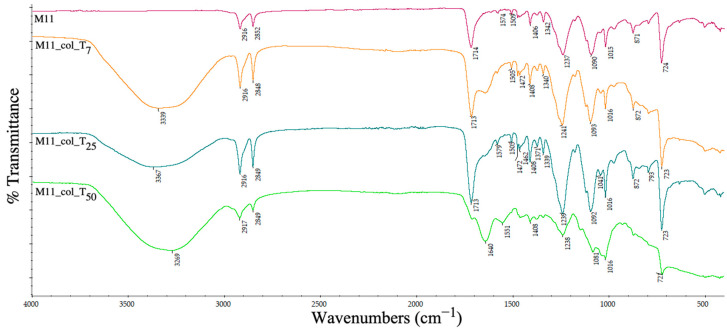
ATR spectra of PET–HDPE composite pellets before and after collagen deposition at days 7 (T_7_), 25 (T_25_) and 50 (T_50_).

The absorption vibrational bands [15] that characterize HDPE can be visualized in Figure 23 (sample M11) with the appearance of surface functional groups located at 2916 cm^−1^ and 2852 cm^−1^ corresponding to the CH_2_– stretching vibration and at 724 cm^−1^ attributed to the CH_2_ rocking mode of the CH_2_ groups. As expected, HDPE signals are overlapped with the PET peaks. The collagen absorption bands were located similarly to the previous spectra (absorption of amides I and II at 1640 cm^−1^), with an increased intensity and conformation based on the immersion period of the pellet sample into the contaminant solution.

**Figure 24:**
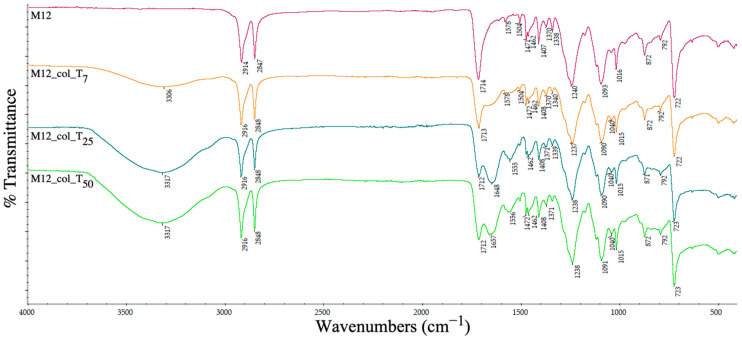
ATR spectra of PET–HDPE–Al nano-power composite pellets before and after collagen deposition at days 7 (T_7_), 25 (T_25_) and 50 (T_50_).

**Figure 25:**
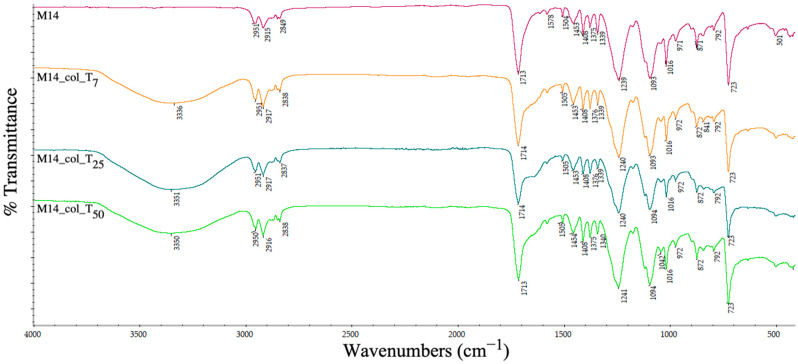
ATR spectra of PET–HDPE–Fe nano-powder composite pellets before and after collagen deposition at days 7 (T_7_), 25 (T_25_) and 50 (T_50_).

In agreement with the swelling degree for PET–HDPE–Al nano-power (5%) and PET–HDPE–Fe nano-powder composites (%), the ATR spectra of the samples (sample M12, Figure 24 and sample M14, Figure 25) showed a different pattern for collagen adsorption. An increased level of collagen was bound to the surface of the PET–HDPE–Al nano-power based pellets, as denoted by the stretching vibrations of the OH and NH bonds of amide A in the region 3306–3317 cm^−1^ and the absorption of new appeared amides I and II at 1657 cm^−1^, even after 7 days of direct contact. 

A slightly different behaviour pattern was observed for the samples containing iron nano-powder (M14) when collagen peaks are visible but overlapped over the main peaks of PET and HDPE; however, it is clear that the collagen was retained on the surface (amide A at 3336/3350/3351 cm^−1^ and amide I and II under the 1714 cm^−1^ peak). The results are in contrast with the contact angle values that increase after the interaction with a hydrophilic molecule. These results indicate that, probably, there are some interactions between HDPE, the incorporated metals and the collagen groups.

## 4. Conclusions

Featuring numerous attractive properties, such as ease of processing, flexibility, non-toxicity, excellent mechanical strength and cost-effectiveness, polymers are widely used in many different fields and being part of our modern life. However, these properties are distorted in the recycling process and the blending assets became strongly related to the compatibility between the reused polymers and other encountered additives or by-products. In this study, we have evaluated the ability of recycled PET, PP and HDPE combined with metal nano-powder composite materials to retain protein contaminants after direct contact. The capacity of PET, PP, HDPE, Al and Fe particles to interact with collagen solution was analysed using swelling capacity and contact angle extents. The ability of collagen to form bridges with the different tested surfaces was analysed using ATR spectroscopy. The results showed that recycled PET pellets retain collagen in the first 25 days, as observed by the appearance of the protein specific vibrational peaks. The addition of PP into the PET matrix composition led to a lower capacity for the pellet to retain collagen on its surface due to the non-polarity of the molecule and the uniformity pattern observed on the micrographs. Even so, when Al was added into the composition, collagen connected with the surface as denoted by the visible amide A, I and II on the ATR spectra. A well-defined capacity to retain protein molecules was detected for the PET–HDPE and PET–HDPE–Al-based pellets (the appearance of sharp amide I and II on the ATR spectra at 50 days post-contact), probably due to the interconnected fibre arrangement of the pellet that may contribute to collagen penetration. Therefore, water repellent characteristics of the polymer-based matrix may protect the surface from contamination and moisture absorption; however, the feature is dependent on the composition and surface configuration. 

## 5. Patents

Parts of the results reported in this manuscript have been submitted for a national patent currently under evaluation, application request no. A/00201/19 April 2022.

## Figures and Tables

**Table 1:** Composition and hydrostatic density of recycled PET hybrid substrates (preliminary results for samples M1, M2, M6, M7, M11 and M12 adapted from our previous paper [16]).

Sample Codification	Recycled PET (%)	PP (%)	HDPE (%)	Al Nano-Powder (%)	Fe Nano-Powder (%)	HD (g/cm^3^)
M1	100	0	0	0	0	1.318 ± 0.0004
M2	95			5		1.347 ± 0.0009
M3	92			8		1.382 ± 0.0011
M4	95			0	5	1.317 ± 0.0018
M5	92				8	1.381 ± 0.0004
M6	70	30			0	1.186 ± 0.0016
M7	66.5	28.5		5		1.395 ± 0.2833
M8	64.5	27.5		8		1.207 ± 0.0013
M9	66.5	28.5		0	5	1.306 ± 0.0000
M10	64.5	27.5			8	1.827 ± 0.6088
M11	70	0	30		0	1.180 ± 0.0004
M12	66.6		28.5	5		1.210 ± 0.0000
M13	64.5		27.5	8		1.219 ± 0.0004
M14	66.5		28.5	0	5	1.228 ± 0.0004
M15	64.5		27.5		8	1.318 ± 0.0004

**Table 2:** Processing temperature regimes.

Sample Codification	Temperatures on Heating Zones (°C)				
M1	300	295	290	285	280
M2–M3	260	255	250	245	240
M4–M5	270	265	260	255	250
M6–M10	260	255	250	245	240
M11					
M12–M15	250	245	240	235	230

**Table 3:** The swelling degree percentage at different time-points.

Sample Codification	Q_7_, %	Q_17_, %	Q_21_, %	Q_25_, %	Q_50_, %
M1	0.22	0.32	0.43	0.58	0.65
M2	0.32	0.54	0.76	0.65	0.68
M3	0.38	0.57	0.54	0.75	0.78
M4	0.24	0.48	0.61	0.73	0.73
M5	0.31	0.52	0.72	0.72	0.72
M6	0.24	0.59	1.06	1.42	1.53
M7	0.39	1.04	1.68	2.07	2.07
M8	0.74	1.89	2.03	2.16	2.30
M9	0.52	1.42	1.94	2.10	2.22
M10	0.69	0.69	1.15	1.61	1.72
M11	0.25	0.88	1.01	1.01	1.01
M12	0.41	2.04	2.99	3.66	3.80
M13	0.50	0.74	0.99	1.12	1.24
M14	3.61	4.48	4.61	6.10	6.10
M15	1.25	1.25	1.25	1.25	1.25

**Table 4:** Contact angle of PET composites pellets before (T_0_) and after 50 days of collagen immersion (T_50_).

Sample Codification	Contact Angle, ° ± SD	
	T_0_	T_50_
M1	95 ± 9	97.5 ± 7.7
M2	99.9 ± 12.6	114 ± 7.1
M3	96.4 ± 5.5	109.3 ±0.42
M4	97.5 ± 3.3	104.5 ± 6.3
M5	94.3 ± 6.6	102 ± 5.6
M6	114 ± 0.1	93.5 ± 3.5
M7	103.9 ± 3.1	100.5 ± 3.5
M8	108.3 ± 0.9	107 ± 15.5
M9	105.5 ± 2.3	97.5 ± 2.12
M10	107.4 ± 14.2	89.5 ± 0.7
M11	114.9 ± 8.6	106 ± 2.7
M12	102.9 ± 1.9	109 ± 5.6
M13	118.1 ± 4.8	107.5 ± 0.7
M14	103. 7 ± 3.7	116 ± 11.3
M15	101.1 ± 6.3	106.5 ± 0.7

## Data Availability

Not applicable.
